# Characterization of attaching and effacing *Escherichia coli *(AEEC) isolated from pigs and sheep

**DOI:** 10.1186/1471-2180-8-144

**Published:** 2008-09-11

**Authors:** Erik Fröhlicher, Gladys Krause, Claudio Zweifel, Lothar Beutin, Roger Stephan

**Affiliations:** 1 Institute for Food Safety and Hygiene, Vetsuisse Faculty University of Zurich, Winterthurerstrasse 272, 8057 Zurich, Switzerland; 2 National Reference Laboratory for *Escherichia coli*, Centre for Infectiology and Pathogen Characterization, Federal Institute for Risk Assessment, Berlin, Germany

## Abstract

**Background:**

Attaching and effacing *Escherichia coli *(AEEC) are characterized by their ability to cause attaching-and-effacing (A/E) lesions in the gut mucosa of human and animal hosts leading to diarrhoea. The genetic determinants for the production of A/E lesions are located on the locus of enterocyte effacement (LEE), a pathogenicity island that also contains the genes encoding intimin (*eae*). This study reports data on the occurrence of *eae *positive *E. coli *carried by healthy pigs and sheep at the point of slaughter, and on serotypes, intimin variants, and further virulence factors of isolated AEEC strains.

**Results:**

Faecal samples from 198 finished pigs and 279 sheep were examined at slaughter. The proportion of *eae *positive samples was 89% for pigs and 55% for sheep. By colony dot-blot hybridization, AEEC were isolated from 50 and 53 randomly selected porcine and ovine samples and further characterized. Strains of the serotypes O2:H40, O3:H8 and O26:H11 were found in both pigs and sheep. In pigs O2:H40, O2:H49, O108:H9, O145:H28 and in sheep O2:H40, O26:H11, O70:H40, O146:H21 were the most prevalent serotypes among typable strains. Eleven different intimin types were detected, whereas γ2/θ was the most frequent, followed by β1, ε and γ1. All but two ovine strains tested negative for the genes encoding Shiga toxins. All strains tested negative for the *bfp*A gene and the EAF plasmid. EAST1 (*ast*A) was present in 18 of the isolated strains.

**Conclusion:**

Our data show that pigs and sheep are a source of serologically and genetically diverse intimin-harbouring *E. coli *strains. Most of the strains show characteristics of atypical enteropathogenic *E. coli*. Nevertheless, there are *stx*-negative AEEC strains belonging to serotypes and intimin types that are associated with classical enterohaemorrhagic *E. coli *strains (O26:H11, β1; O145:H28, γ1).

## Background

Attaching and effacing *Escherichia coli *(AEEC) are characterized by their ability to cause attaching-and-effacing (A/E) lesions in the gut mucosa of human and animal hosts leading to diarrhoea. The main mechanism of AEEC pathogenesis is the destruction of the gastric microvillus brush border through restructuring of the underlying cytoskeleton by signal transduction between bacterial and host cells, intimate adherence of strains to the intestinal epithelium, pedestal formation and aggregation of polymerized actin at the sites of bacterial attachment [1,2]. The adherence of bacteria to the enterocytes is mediated by intimin, an outer membrane protein encoded by the *eae *(*E. coli *attachment effacement) gene [2].

Intimin genes are present in enteropathogenic *E. coli *(EPEC) and some Shigatoxin-producing *E. coli *(STEC). EPEC strains are defined as *eae *harbouring diarrhoeagenic *E. coli *that possess the ability to form A/E lesions on intestinal cells and that do not possess Shigatoxin encoding genes [3]. According to their virulence markers, EPEC strains are subdivided into typical and atypical EPEC. Typical EPEC harbour the EAF (EPEC adherence factor) plasmid, which carries genes for regulation of LEE functions and for production of bundle-forming pili (BFP), which interconnect bacteria within microcolonies and lead to a characteristic localized adherence pattern. Atypical EPEC strains are negative for both, the EAF plasmid and BFP and show diffuse, aggregative or localized-like adherence patterns [2,4]. Moreover, typical and atypical EPEC usually belong to certain serotype cluster and differ in their geographic distribution and their natural reservoir. Typical EPEC are still a major cause of infantile diarrhoea in developing countries and are rarely found in animals. Atypical EPEC strains predominate in industrialized countries and can be isolated from both humans and animals [4]. Atypical EPEC appear to be more closely related to STEC and as such are considered emerging pathogens [1,4,5]. Their role in human infections is probably underestimated.

The second group of AEEC is formed by STEC strains, which produce Shigatoxins. STEC are responsible for a number of human gastrointestinal diseases, including diarrhoea and hemorrhagic colitis (HC). In a proportion of individuals, particularly in children, these conditions may be complicated by neurological and renal sequelae, including haemolytic-uremic syndrome (HUS) [6].

Although a number of studies have looked for the *eae *gene in STEC strains isolated from sheep and pigs, only a limited number of studies have been undertaken to screen pigs and sheep for AEEC and to further characterize such strains [7-9].

The aim of this study was to isolate *eae *positive *E. coli *strains carried by healthy pigs and sheep at slaughterhouse level and to provide further characterization data for such strains.

## Results

A total of 198 faecal samples from pigs and 279 faecal samples from sheep collected at slaughter were tested for the presence of *eae *positive *E. coli*. AEEC were shed by 176 (89%) and 154 (55%) of pigs and sheep, respectively. Using colony dot-blot hybridization, 50 porcine and 53 ovine AEEC strains were identified and isolated.

Twenty-seven of the 50 *eae *positive porcine *E. coli *strains were typeable with O antisera (Table 1). They belonged to ten O serogroups, whereas 17 strains were of three serogroups, namely O2 (10 strains), O145 (4 strains) and O108 (3 strains). Eighteen strains were not typeable for the O-antigen (ONT) and five had a rough Lipopolysaccharide (LPS type, spontaneously agglutinating). Strains of the serogroup O2 showed different H types and the two strains of serotype O88:H25 carried two different intimin variants (η and ζ). Thirty-six of the 53 *eae *positive ovine *E. coli *strains were typeable with O antisera (Table 2). They belonged to 15 O serogroups, whereas 17 strains were of three serogroups, namely O146 (9 strains), O2 (4 strains) and O70 (4 strains). Sixteen strains were ONT and one had a rough LPS type. Strains of the serogroup O70 and O116 showed different H types. Overall, only five O26:H11 strains (two porcine and three ovine strains) belonged to a known human EPEC serotype. Besides, this serotype (O26:H11) has also been reported in association with human enterohaemorrhagic *E. coli *(EHEC) strains causing severe disease.

**Table 1:** Characteristics of 50 attaching and effacing *Escherichia coli *isolated from pigs at slaughter (n = 50)

Serotype	No. of strains	*eae *type	*ast*A	*tir *genotype	*stx*
O2:H8	1	γ2/θ	-	Y-P	-
O2:H40	3	γ2/θ	-	Y-P	-
O2:H49	4	ι	-	Y-P	-
O2:HNT	1	γ2/θ	-	Y-P	-
O2:HNT	1	γ2/θ	-	Y-P	-
O3:H8	1	γ2/θ	-	Y-P	-
O26:H11	2	ε	-	Y-P	-
O45:H11	1	β1	-	Y-P	-
O88:H25	1	η	-	Y-P	-
O88:H25	1	ζ	-	Y-P	-
O108:H9	3	β1	-	Y-P	-
O121:H45	1	γ2/θ	-	Y-P	-
O145:H28	4	γ1	-	S	-
O153:H10	1	κ	-	Y-P	-
O153:HNT	1	κ	+	Y-P	-
O180:H2	1	ε	-	untypable	-
ONT:H2	2	β1	-	Y-P	-
ONT:H2	3	ε	-	Y-P	-
ONT:H5	2	κ	-	Y-P	-
ONT:H6	1	α2	+	S	-
ONT:H6	1	γ2/θ	-	Y-P	-
ONT:H7	4	γ2/θ	-	Y-P	-
ONT:H10	2	κ	+	Y-P	-
ONT:H11	1	β1	-	Y-P	-
ONT:H40	1	γ2/θ	-	Y-P	-
ONT:H49	1	ι	-	Y-P	-
Orough:H11	1	β1	-	Y-P	-
Orough:H28	2	γ1	-	S	-
Orough:H40	1	γ2/θ	-	Y-P	-
Orough:HNT	1	γ1	-	S	-

Y-P: EPEC-type translocated intimin receptor

S: STEC-type translocated intimin receptor

*eae*: gene encoding intimin

*ast*A: gene encoding EAST1

*tir*: gene encoding translocated intimin receptor

*stx*: gene encoding Shigatoxin

**Table 2:** Characteristics of 53 attaching and effacing *Escherichia coli *isolated from sheep at slaughter (n = 53)

serotype	No. of strains	*eae *type	*ast*A	*tir *genotype	*stx*
O2:H40	3	γ2/θ	-	Y-P	-
O2:H40	1	γ2/θ	+	Y-P	-
O3:H8	1	γ2/θ	-	Y-P	-
O26:H11	3	β1	-	Y-P	-
O35:H2	1	β1	-	Y-P	-
O51:H49	1	α1	-	Y-P	-
O70:H11	1	β1	-	Y-P	-
O70:H40	3	γ2/θ	+	Y-P	-
O71:H11	1	β1	-	Y-P	-
O96:H7	1	β2/δ	-	Y-P	-
O103:H25	1	γ2/θ	-	Y-P	-
O116:H5	1	β1	-	Y-P	-
O116:H20	2	β1	-	Y-P	-
O121:H19	2	ε	-	Y-P	+
O145:H34	1	ι	-	Y-P	-
O145:HNT	1	γ1	-	S	-
O146:H21	5	γ2/θ	+	Y-P	-
O146:H21	4	γ2/θ	-	Y-P	-
O156:HNT	1	ζ	-	Y-P	-
O156:HNT	1	γ2/θ	-	Y-P	-
O161:HNT	1	ε	-	Y-P	-
ONT:H2	2	β1	-	Y-P	-
ONT:H6	1	α1	-	Y-P	-
ONT:H6	1	β2/δ	+	Y-P	-
ONT:H6	2	β2/δ	-	Y-P	-
ONT:H6	1	untypable	-	S	-
ONT:H9	1	β1	+	Y-P	-
ONT:H31	1	γ2/θ	-	Y-P	-
ONT:H40	2	γ2/θ	-	Y-P	-
ONT:HNT	1	γ1	+	S	-
ONT:HNT	1	γ2/θ	-	Y-P	-
ONT:HNT	1	κ	+	Y-P	-
ONT:HNT	1	β1	-	Y-P	-
ONT:HNT	1	ε	-	Y-P	-
Orough:H6	1	β2/δ	+	Y-P	-

Y-P: EPEC-type translocated intimin receptor

S: STEC-type translocated intimin receptor

*eae*: gene encoding intimin

*ast*A: gene encoding EAST1

*tir*: gene encoding translocated intimin receptor

*stx*: gene encoding Shigatoxin

In total, a variety of 11 different intimin types and subtypes were found. The 50 isolated porcine AEEC (Table 1) harboured 9 variants, and β1 (8 strains), γ1 (7 strains), ε (6 strains), γ2/θ (15 strains), ι (5 strains), κ (6 strains) were the frequently found. The 53 isolated ovine *eae *positive *E. coli *strains (Table 2) harboured 9 different intimin variants, and β1 (13 strains), β2/δ (5 strains),ε (4 strains), γ2/θ (23 strains) were the most frequently found. One strain was not typeable. For this strain no PCR product was obtained with SK1 as the forward primer and all used reverse primers.

All but two ovine AEEC strains (O121:H19, intimin ε) tested negative for genes encoding Shigatoxins. All strains tested negative for *bfp*A and the EAF plasmid. EAST1 (*ast*A) was present in 18 strains, four porcine and 14 ovine strains. Forty-one porcine and 50 ovine strains tested positive for *tir*-Y-P (EPEC-type called Y-P). For one porcine strain there were no PCR products present in the *tir *specific PCRs. This strain was therefore not typable for this genotype. Remarkably, all strains encoding intimin γ1 were associated with *tir*-S (STEC-type called S).

## Discussion

The high percentages of animals shedding AEEC are remarkable, especially in view of frequencies of sheep and pigs colonized with AEEC found in these animal species in previous works [7-9]. Indeed, de la Fuente et al. [7] and Krause et al. [9] detected AEEC in less than 20% of the sheep and pigs studied, while in our work these percentages were 89% for pigs and 55% for sheep. Aktan et al. [8] found 17.7% and 0.75% AEEC strains from sheep and pigs, respectively.

In our study, no strains showing characteristics of typical EPEC were found. These results are consistent with earlier reports that most AEEC strains from sheep and pigs are negative for *stx *genes and *bfp*A, and are therefore considered as atypical EPEC [8,9].

Pigs and sheep are reservoirs of serologically and genetically diverse intimin harbouring *E. coli*. In our study, only strains of the serotypes O2:H40, O3:H8 and O26:H11 were commonly isolated from both pigs and sheep. All other serotypes were different. This could indicate that atypical EPEC are adapted to their particular animal host. In pigs, serogroups O2, O108 and O145 and serotypes O2:H40, O2:H49, O108:H9, O145:H28 were most frequently found. Intimin types showed a wide diversity with θ, β1 and γ1 as the most frequent types. These results partly agree with other studies performed on healthy pigs in Germany and Hungary [9,10], considering that the latter study was performed on weaned pigs. Our results show more intimin types and slightly different serotype patterns with more untypeable O serogroups and O2 as the most frequent one. In sheep, serogroups O2, O26; O70 and O146 and serotypes O2:H40, O26:H11, O70:H40, O146:H21 were most frequently found. Intimin types showed a wide diversity with γ2/θ and β1 as the most frequent types. Compared to studies performed on healthy sheep in England, Wales, and Australia [8,11], the ovine strains isolated in Switzerland harboured more frequently the intimin γ2/θ type. But a high prevalence (37.5%) of this intimin type in healthy sheep was also shown in a Spanish study [12].

Ovine isolates reported in the literature show more genetic similarity to bovine than porcine strains. Namely O2, O26, O35, O70, O103, O145 and O156 were found in studies on healthy cattle in Germany, England and Wales, Spain, Switzerland and Brazil [8,9,12-14]. Apart from intimin γ2/θ, which was found much more often in ovine strains than bovine, the distribution of intimin types is rather similar in the present study.

The only classical human EPEC serotype found in our study was O26:H11 (2 pig strains, 3 ovine strains), indicating that classical EPEC serotypes might not be very common in these animal species. Moreover, no serotypes known for causing large outbreaks such as O39:H-, O88:H55, O91:H7 and O111:H9 were found. However, 17% of all strains carried EAST1. The percentage (26.4%) of ovine AEEC strains that harboured this gene was similar to that found by Aktan et al. [8], but much higher than that found by Yuste et al. [15]. Even the significance of this low-molecular-weight, plasmid-mediated, heat-stable enterotoxin in AEEC pathogenesis is still unknown, large EPEC outbreaks in adults have been associated with EAST1 positive strains and recent studies found EAST1 to be associated significantly with diarrhoea [16-18].

Hence most of the strains showed characteristics of atypical EPEC. Nevertheless, there are seven *stx *negative AEEC strains belonging to serotypes and *eae *types that are associated with typical EHEC strains (O26:H11, β1; O145:H28, γ1). The reason that these strains are *stx *negative might be due to the fact that STEC strains can undergo ephemeral interconversions via loss and gain of Stx-encoding phages, which leads to different pathotypes. This was already shown for *E. coli *O157 of the H7 clone [19,20] and for *E. coli *O26 [21]. Ongoing studies in our laboratories aim to further characterize the O26 and O145 strains in view of phage integration sites as well as to perform transduction experiments on these strains.

## Conclusion

Our data show that pigs and sheep are a source of serologically and genetically diverse intimin harbouring *E. coli *strains. Most of the strains show characteristics of atypical EPEC. Nevertheless, there are *stx *negative AEEC belonging to serotypes and intimin types that are associated with typical EHEC strains (O26:H11, β1; O145:H28, γ1).

## Methods

### Strains

Samples examined in this study were collected within nine months (from March to November 2007) in an EU-approved slaughterhouse in Switzerland. In total, fecal samples of 198 healthy finished pigs and 279 healthy adult sheep randomly distributed over Switzerland were collected during 10 and 11 sampling days, respectively. Samples were subsequently placed into cool boxes. Microbiological examination was carried out within 1 to 3 h after sampling. Upon arrival in the laboratory, 10 g of each sample were enriched in 100 ml of brilliant green bile broth (BBL, Cockeysville, Md.) at 37°C for 24 h.

The enriched samples were streaken onto sheep blood agar (Difco Laboratories, Detroit, Mich.; 5% sheep blood Oxoid, Hampshire, UK), and after incubation at 37°C for another 24 h, the colonies were washed off with 2 ml of 0.85% saline solution. Of each plate eluate 2.0 μl were then evaluated by PCR with primers SK1 and SK2 amplifiying a 863-kb product targeting sequences at the 5' *eae *conserved region detecting all types of *eae *described at the moment [22]. From the *eae *PCR-positive samples, 50 porcine and 53 ovine samples were randomly selected for strain isolation with an *eae *DNA probe and colony dot-blot hybridization. The *eae *probes were prepared by labelling *eae*-PCR amplicons from *E. coli *O157:H7 strain 857/03 with DIG High Prime kit (Roche, Mannheim, Germany). For colony hybridization, the *eae *positive samples were plated onto sheep blood agar and incubated overnight at 37°C. Colonies were transferred to a nylon membrane (Roche), and lysed following standard methods. After washing, crosslinking, and prehybridization in DIG-Easy-Hyb buffer (Roche) at 42°C for about 60 min, hybridization of membranes with *eae *DNA probes was performed overnight at 42°C. After washing in primary and secondary wash buffers, the presence of labelled probe was detected with an alkaline phosphatase-conjugated antibody detection kit and NBT/BCIP stock solution according to the instructions of the manufacturer (Roche). Positive colonies were picked from the original sheep blood agar and confirmed to be *eae *positive by PCR. One randomly chosen colony per sample was used for further strain characterization.

### Strain characterization

Determination of O antigens was performed as described [23] by tube agglutination tests with boiled cultures of bacteria using monospecific O-antisera (O1 to O185) at the National Reference Laboratory for *Escherichia coli *(NRL-*E. coli)*. Determination of H-types was performed with H-specific antisera prepared at the NRL-*E. coli *(BfR) and flagellar (*fliC*) genotypes of nonmotile strains were identified by PCR followed by digestion of PCR products with *Hha*I resulting in *fliC*-genotype-specific profiles as described previously [24].

Subtyping of intimin genes was performed using primer SK1 in combination with intimin-type specific reverse primers [22,25]. Genetic subtyping of *eae *genes encoding intimins α, β and γ was done by restriction fragment length polymorphism (RFLP)-analysis of *PstI *digested PCR products as described [22]. For detection of further putative virulence genes, strains were examined for the presence of *stx *genes, the EAF plasmid, the *bfp*A gene, and the *ast*A gene encoding EAST1 (Table 3). To distinguish between phosphorylated *tir*_E2348/68 _(EPEC-type called Y-P) and nonphosphorylated *tir*_Sakai _(STEC-type called S), gene specific forward primers (tirY474-F: 5'-CATATTTATGATGAGGTCGCTC-3' and tirS478-F: 5'-TCTGTTCAGAATATGGGGAATA-3') were used together with a conserved reverse primer (tirR: 5'-TAAAAGTTCAGATCTTGATGACAT-3') [26]. In order to improve the results in this PCR (5 min 94°C; 34 × 30 sec 94°C, 30 sec 50°C, 30 sec 72°C; 5 min 72°C), MgCl2 was added to a final Mg^2+ ^concentration of 5 mM. All PCR reactions were run with a negative and a positive control strain.

**Table 3:** PCR primers used for the identification and characterization of attaching and effacing *Escherichia coli *strains

Target	Primer	Oligonucleotide sequence (5'-3')	Reference
*astA*	East11a	CCA TCA ACA CAG TAT ATC CGA	27
	East11b	GGT CGC GAG TGA CGG CTT TGT	
*bfpA*	EP1	AAT GGT GCT TGC GCT TGC TGC	5
	EP2	GCC GCT TTA TCC AAC CTG GTA	
*eae*	SK1	CCC GAA TTC GGC ACA AGC ATA AGC	22
	SK2	CCC GGA TCC GTC TCG CCA GTA TTC G	
*eae-*α	SK1-LP2	CCC GAA TTC TTA TTT TAC ACA AGT GGC	25
*eae-*γ	SK1-LP3	CCC GAA TTC TTC TTT TAC ACA AAC CGC	25
*eae-*β	SK1-LP4	CCC GTG ATA CCA GTA CCA ATT ACG GTC	22
*eae-*ε	SK1-LP5	AGC TCA CTC GTA GAT GAC GGC AAG CG	22
*eae-*ζ	SK1-LP6B	TAG TTG TAC TCC CCT TAT CCC	25
*eae-*ι	SK1-LP7	TTT ATC CTG CTC CGT TTG CT	25
*eae-η*	SK1-LP8	TAG ATG ACG GTA GAC	25
*eae-κ*	SK1-LP10	GGC ATT GTT ATC TGT TGT CT	25
*eae-θ*	SK1-LP11B	GTT GAT AAC TCC TGA TAT TTT A	25
EAF	EAF1	CAG GGT AAA AGA AAG ATG ATA A	27
	EAF25	TAT GGG GAC CAT GTA TTA TCA	
*fliC*	FliC up	CAA GTC ATT ATT AC(AC) AAC AGC C	5
	FliC down	GAC AT(AG) TT(AG)GA(AGC) ACT TC(GC) GT	
*stx*	VT1	ATT GAG CAA AAT AAT TTA TAT GTG	27
	VT2	TGA TGA TGG CAA TTC AGT AT	
*tir*	*tir*-R	TAA AAG TTC AGA TCT TGA CAT	26
*tir *Y-P	tirY474-F	CAT ATT TAT GAT GAG GTC GCT C	
*tir *S	tisS478-F	TCT GTT CAG AAT ATG GGG AAT A	

*ast*A: gene encoding EAST1

*bfp*A: gene encoding bundle-forming pili

*eae*: gene encoding intimin

*fliC*: gene encoding flagellum

EAF: EPEC adherence factor

*tir*: gene encoding translocated intimin receptor

*stx*: gene encoding Shigatoxin

## Authors' contributions

RS and CZ designed the study and drafted the manuscript. EF isolated the strains and was responsible for further strain characterisation, GK and LB performed serotyping of the strains and the PCR typing of *eae *genes. All authors read, commented on and approved of the final manuscript.
